# Antimicrobial resistance in Antarctica: is it still a pristine environment?

**DOI:** 10.1186/s40168-022-01250-x

**Published:** 2022-05-06

**Authors:** K. Hwengwere, H. Paramel Nair, K. A. Hughes, L. S. Peck, M. S. Clark, C. A. Walker

**Affiliations:** 1 School of Life Sciences, Faculty of Science and Engineering, Anglia Ruskin University, East Road, Cambridge, CB1 1PT UK; 2 Marine Biology and Ecology Research Centre, School of Biological and Marine Sciences, University of Plymouth, Drake Circus, Plymouth, PL4 8AA UK; 3 British Antarctic Survey, Natural Environment Research Council, High Cross, Madingley Road, Cambridge, CB3 0ET UK

**Keywords:** β-Lactam, Efflux pumps, Aminoglycosides, Horizontal gene transfer, Wastewater treatment, Metagenomics, Anthropogenic, Sewage

## Abstract

Although the rapid spread of antimicrobial resistance (AMR), particularly in relation to clinical settings, is causing concern in many regions of the globe, remote, extreme environments, such as Antarctica, are thought to be relatively free from the negative impact of human activities. In fact, Antarctica is often perceived as the last pristine continent on Earth. Such remote regions, which are assumed to have very low levels of AMR due to limited human activity, represent potential model environments to understand the mechanisms and interactions underpinning the early stages of evolution, de novo development, acquisition and transmission of AMR. Antarctica, with its defined zones of human colonisation (centred around scientific research stations) and large populations of migratory birds and animals, also has great potential with regard to mapping and understanding the spread of early-stage zoonotic interactions. However, to date, studies of AMR in Antarctica are limited. Here, we survey the current literature focussing on the following:

i) Dissection of human-introduced AMR versus naturally occurring AMR, based on the premise that multiple drug resistance and resistance to synthetic antibiotics not yet found in nature are the results of human contamination
ii) The potential role of endemic wildlife in AMR spread

There is clear evidence for greater concentrations of AMR around research stations, and although data show reverse zoonosis of the characteristic human gut bacteria to endemic wildlife, AMR within birds and seals appears to be very low, albeit on limited samplings. Furthermore, areas where there is little, to no, human activity still appear to be free from anthropogenically introduced AMR. However, a comprehensive assessment of AMR levels in Antarctica is virtually impossible on current data due to the wide variation in reporting standards and methodologies used and poor geographical coverage. Thus, future studies should engage directly with policymakers to promote the implementation of continent-wide AMR reporting standards. The development of such standards alongside a centralised reporting system would provide baseline data to feedback directly into wastewater treatment policies for the Antarctic Treaty Area to help preserve this relatively pristine environment.

Video Abstract

**Supplementary Information:**

The online version contains supplementary material available at 10.1186/s40168-022-01250-x.

## Introduction

Antimicrobial resistance (AMR) is a major global health problem [1]. The highly influential O’Neill report predicted that AMR could cost the global economy billions of dollars and the lives of 10 million people, every year by 2050 [2], figures which will almost certainly be further exacerbated by the effects of SARS-CoV2 [3]. Thus, concerted global action is required to tackle this problem from many different angles. The O’Neill report identified ten critical interventions, one of which was surveillance [2]. Whilst fundamental for tracking the emergence and spread of AMR, the development of effective policies and interventions ultimately depends on surveillance [4]. Indeed, global AMR surveillance has begun identifying weaknesses in national systems against AMR and producing evidence for important drivers of AMR spread [5]. Nonetheless, surveillance studies are limited by the quality and formatting of data. Global surveillance systems are disjointed, sampling numbers are low, methodologies for surveillance are not uniform and not all clinical data are recorded electronically [6].

One global region where surveillance is particularly poor is Antarctica. This remote, extreme environment is not a known AMR hotspot, yet relatively pristine regions, where levels of human settlement are low, can act as simpler model systems to study the factors that propagate AMR. Investigating AMR in such regions can help determine the extent to which human activity has promoted the spread of antimicrobial resistance genes and antibiotic-resistant bacteria [7]. In addition, Antarctica is home to many species of charismatic megafauna (e.g. whales, seals, penguins, albatrosses), which migrate long distances, including to regions with large human populations [8, 9]. These animals have the potential to harbour human-associated antibiotic-resistant bacteria and act as vectors, as previously demonstrated in Arctic terns [10]. Hence, Antarctica also provides a useful model to dissect the role of migratory wildlife and their interaction with humans to understand the increasing anthropogenic influence on the spread of AMR [11].

Antarctica is known as the last pristine continent, effectively isolated from the rest of the world by both oceanic and atmospheric circulations [12, 13]. Although it is a difficult-to-reach and extremely cold environment, human presence in Antarctica has gradually increased since the first recorded landings in the nineteenth century [14, 15]. Today, this is large due to the generally expanding tourism industry (with over 74,000 visitors to the region during the 2019/2020 summer season, albeit numbers fell dramatically with the commencement of the COVID pandemic [16]) and the increasing presence and activity of governmental Antarctic programmes [17, 18]. Although the annual number of tourists generally far outweighs the yearly number of staff of national programmes operating via Antarctic research stations (around 5000), the latter cumulatively spend much longer on the continent, often several months per scientist. Such scientific visits may comprise station-based science, but also include many other ship- and aircraft-based activities, including deployment and supply of field parties. Tourists are generally on short trips of a couple of weeks and based on cruise ships that generally facilitate brief landings at a small number of easily accessible locations and either take sewage out of Antarctica or, at a minimum, dispose of it at least 12 nautical miles from the coast [19, 20]. Thus, occupancy of research stations, one of which has in excess of 1000 people living there in the summer, represents the higher risk for the spread of AMR on the continent, compared with tourists, as sewage is largely dealt with on the continent. As will be described later, the treatment of sewage is highly variable between different research stations.

Historically, the rapid rise of human presence in Antarctica only started in the build-up to the International Geophysical Year in 1957/1958. Before that, there was relatively little human activity apart from a couple of whaling stations and Argentinian and UK research stations (Orcadas Base established on Laurie Island, South Orkneys in 1903 and several stations along the Antarctic Peninsula resulting from Operation Tabarin in 1943, respectively). The International Geophysical Year (IGY) of 1957-1958 sponsored the building of several research stations in Antarctica to support scientific exploration. This resulted in the core of the present-day network, which currently comprises 30 nations operating 76 permanent research stations [21, 22]. These represent a range of different sized operations, with some stations operating all year round, whilst others are only open for a few months in the Austral summer. Most stations generally have fewer than 60 beds. A handful has over 100 beds, including Marambio (170), Frei and Amundsen-Scott Pole (with 150 beds apiece), Rothera (136), Syowa (130) and Mario Zucchelli (124), but all are dwarfed by McMurdo, run by the USA near the Ross Sea with a capacity for 1200 personnel. Critically, the initial period of the station building and scientific expansion in Antarctica coincided with the development of modern antibiotics. Thus, even the earliest scientists had access to sulfonamides, as evidenced by medical stores lists from 1949 showing tablets and bottles of sulphathiazole (Additional file 1). They also potentially had access to penicillin in a period when waste regulation was non-existent.

Uniquely, Antarctica, more particularly all regions from 60° S latitude to the South Pole, is governed through consensus by the 29 Consultative Parties to the Antarctic Treaty (see https://www.ats.aq). The Treaty which designated Antarctica ‘as a natural reserve, devoted to peace and science’, includes the Protocol on Environmental Protection to the Antarctic Treaty (1991) and puts in place strict regulations for environmental protection, including waste management, although the requirements for wastewater treatment are limited (see https://www.ats.aq/e/protocol.html). To date, there have been no rigorous evaluations of AMR in Antarctica, which clearly has health implications for personnel working in such a remote environment, although this issue has been noted by the Council of Managers of National Antarctic Programs [23].

Here, we conduct an extensive review of published studies of AMR, south of 60° S identifying antibiotic-resistant bacteria, antibiotic-resistant genes or resistance-associated plasmids. The criteria for literature screening and inclusion in this review are presented in Additional file 2. It should be noted that studies specifically targeted at identifying novel antimicrobials in Antarctic bacteria for biotechnology exploitation are excluded, as they do not represent the main focus of this review. We present a synthesis of findings, evaluating the extent of resistance to semi-synthetic and synthetic antibiotics (often used as a proxy for anthropogenic-introduced AMR) and the efficacy of methods of surveillance and relate these to potential future policy decisions.

### AMR in Antarctica

**Fig. 1:**
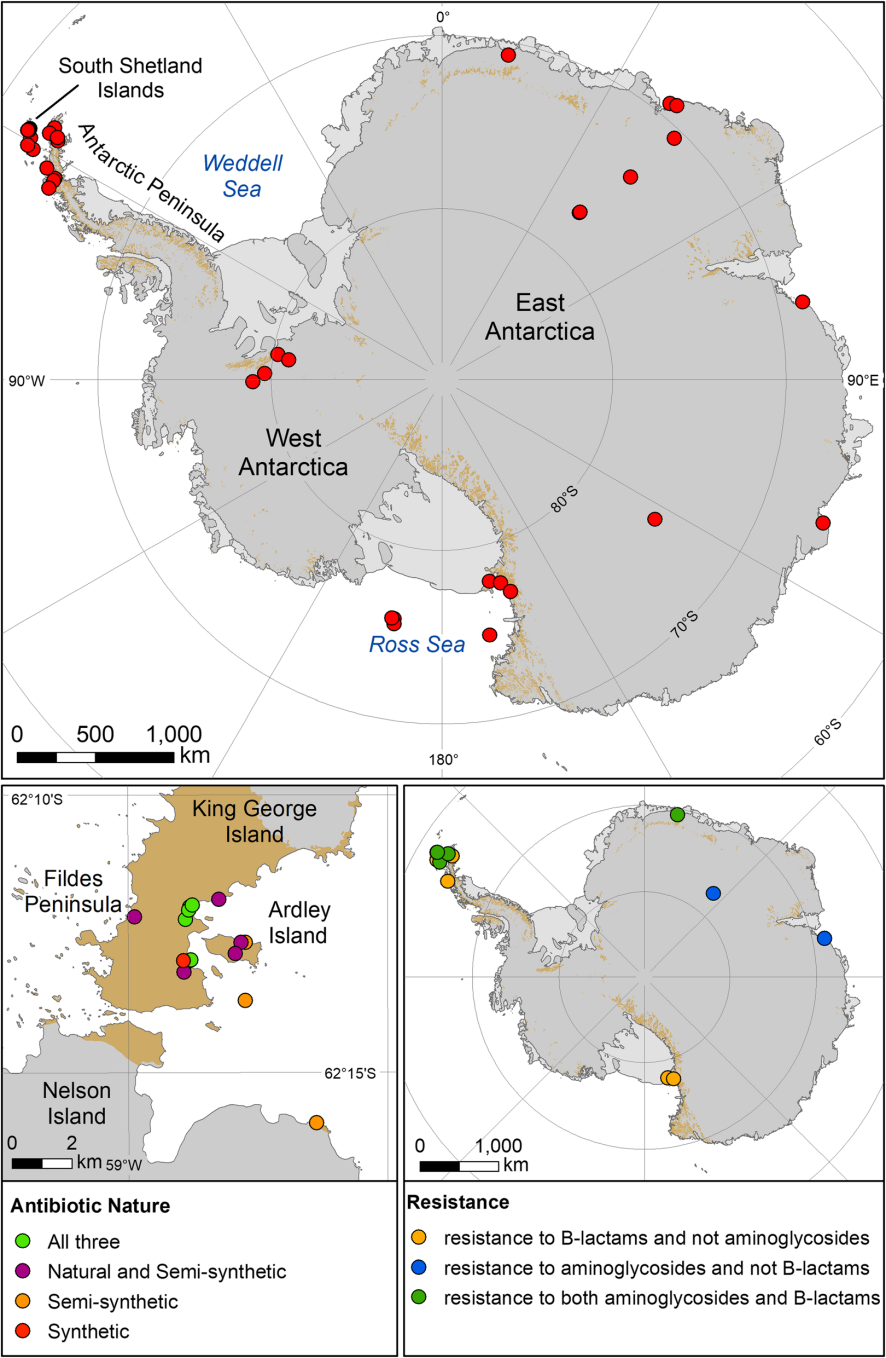
Top panel: map of Antarctica showing the locations in which all the studies analysed in this review took place. If a single author investigated more than one site and clearly distinguished the results of each site, each sample site was recorded individually in Additional file 3. Markers in the sea are from cruise samplings. Bottom left panel: summary of studies conducted in the South Shetland Islands according to the types of antibiotic identified at each site; full details of studies are detailed in Additional file 4. Bottom right panel: summary of resistance found to β-lactams and aminoglycosides in Antarctica; full details of studies are detailed in Additional file 5. NB: when an antibiotic class is annotated as not being present, e.g. resistance to β-lactams not aminoglycosides, this means that the relevant study did not find resistance to aminoglycosides, rather than it being absent

Today, a wide range of antibiotics are available, which work against different aspects of cellular biosynthesis. These can be designated as natural, semi-synthetic or synthetic (Additional file 6), which aid in identifying whether humans are the potential drivers of resistance. Research investigating AMR in Antarctica dates back to the late 1970s/early 1980s with the limited screening (mainly for penicillin and tetracycline resistance) of environmentally isolated Antarctic bacteria and plasmids [24–26]. Since then, AMR screening on the continent has increased and revealed interesting results on the natural levels of AMR, mechanisms of resistance (albeit largely based on the identification of antibiotic resistance genes via sequencing, without associated functional data), evidence for anthropogenic influences on AMR levels and animals as vectors of AMR (Fig. 1, top panel; Additional file 3).

### Endemic AMR

AMR is a natural phenomenon. A few studies have failed to isolate resistance in pristine Antarctic isolates [27–29], but this may simply be due to the methodologies employed in the studies, and antibiotic-resistant genes have been detected at low levels in Antarctic bacteria. Antibiotic-resistant bacteria have been isolated from diverse environmental samples including soils, hypoliths (bacterial communities under rocks), freshwater lakes and glacial ice cores from regions without human activity [30–33]. Whilst the prevalence of human-derived AMR in such pristine and semi-pristine environments is intuitively surprising, it is suggested that it is the extreme nature of the environment that has fuelled resistance, particularly for broad-spectrum resistance [34]. Most conventional antibiotics originate from species within natural environments as a component of normal defence mechanisms. In harsh environments, bacteria compete for limited nutrients, and the ability to produce antibiotics would provide a competitive advantage within these restricted and slow-growing communities [34]. For example, the prevalence of bacterial β-lactamases was suggested as a defence mechanism against the β-lactam-producing fungi prevalent in some soil samples [35]. This competitive advantage theory was validated by the study of Van Goethem et al. [32], in which the number of antibiotic-resistant genes in a sequenced environmental sample was negatively correlated with species richness [31–33, 35, 36]. These studies, although limited in extent, are providing important clues as to how the mechanisms of AMR may have evolved in this environment.

Single-strain genome sequencing has identified a high diversity of antibiotic-resistant genes present in Antarctic bacteria. For example, numerous multi-drug efflux pumps were identified in the genome of *Sphingomonas* sp. strain Ant H11 from an Antarctic hypolith [31]. Interestingly, a *sul2-strA* gene cluster encoding resistance to sulphonamides was identified in DNA extracted from a 1200–1400-ybp (years before present) ice core. Sulphonamides are synthetic drugs, but an antibiotic-resistant gene cluster in such an ancient sample supports the hypothesis that most antibiotic-resistant genes active against synthetic antibiotics originated from environmental bacteria [33]. Hence, this gene cluster active against a ‘synthetic antibiotic’ may mimic a natural chemical yet to be discovered/classified. These findings are supported by the identification of diverse antibiotic-resistant genes extracted from 30,000-year-old permafrost sediments [37] and phylogenetic analyses of β-lactamase genes dating their origin to over 2 billion years ago [38]. Both of these Antarctic genome sequencing studies failed to show the presence of mobile genetic elements (e.g. transposons, integrons and recombinases) associated with antibiotic-resistant genes, indicating that either horizontal gene transfer was unlikely to be involved in their propagation or that such mechanisms are not evolutionarily stable (or could be lost in the absence of specific selection pressures). In contrast, a number of mobile genetic elements were found in the genome of *Staphylococcus edaphicus* sp. that encoded a high number of antibiotic-resistant genes including β-lactamase and an alternative penicillin-binding *MecC* protein [35]. However, a more expansive metagenomic shotgun approach showed a complete absence of mobile genetic elements flanking antibiotic-resistant genes leading to the hypothesis that vertical transmission has a much greater role than horizontal gene transfer in the dissemination of antibiotic-resistant genes in pristine Antarctic soils, with phylogeny, rather than horizontal gene transfer, driving resistome content [32]. Furthermore, this latter study indicated that the major resistance mechanism was via single and multidrug efflux pumps (comprising 60% of antibiotic-resistant gene identifications) with antibiotic inhibition strategies, exemplified by β-lactamases, less commonly identified [32, 39]. A more recent molecular study, specifically screening for integrons and antibiotic-resistant genes, identified a considerable number in Antarctic soil samples but failed to show any association between the two [40]. A clearer pattern may emerge with the generation of much needed additional data, as current data are very limited.

The examples above relate to terrestrial samples (hypoliths and soil), but antibiotic-resistant genes have also been found in freshwater lake and marine samples, albeit at much lower levels than soil samples [41, 42]. In a wide-ranging study on bacterium-bacterium interactions amongst psychrophilic bacteria isolated from Terra Nova Bay, only 15% of cultivated bacteria exhibited inhibitory interactions, and even fewer had some level of identifiable AMR [43, 44]. It was suggested that this relatively low level of inhibition, compared with other studies, was due to the fact that the bacteria were isolated from unfiltered seawater and were in a free-living stage, rather than a biofilm or associated with organic particles [45]. Hence, they were not in close proximity with each other and, therefore, did not have to devote unnecessary cellular energy to antagonistic and competitive biochemical interactions [41]. Interestingly antibiotic-resistant gene-containing bacteriophage particles have also been isolated from Antarctic seawater, and again, it is thought that acquisition and maintenance were due to natural community competition [46]. It is known that bacteriophage play a role in the dissemination of antibiotic-resistant genes in aquatic environments, indicating that in this environment, horizontal transfer of antibiotic-resistant genes may be more prevalent [41, 42, 46]. Thus, the two types of environment (terrestrial and marine) may have different evolutionary mechanisms for the gain and maintenance of AMR. In general, the relative abundance of antibiotic-resistant genes in Antarctica is much lower than that in other global regions where there is greater anthropogenic influence [47]. Thus, it has been hypothesised that the mechanisms of resistance found in the pristine Antarctic environments depict a ‘pre-antibiotic’ state or rather a ‘pre-widespread human use of antibiotics in medicine’ state [39].

### Identification of potentially anthropogenically introduced AMR

Globally, the emergence of AMR as a serious health concern is connected to human activity. The increases in human activity on the Antarctic continent have provoked a question of whether humans may be introducing antibiotic-resistant genes into this unique region [10]. However, answering this research question is complicated by the presence of endemic resistance, as detailed above. Whilst a general strategy of sampling soil and water at varying distances to established research stations was adopted to correlate AMR with levels of human activity, researchers have developed different strategies to distinguish human-associated from naturally occurring resistance. For example, mesophiles have been used as a proxy for human-derived bacteria and psychrophiles as a proxy for endemic bacteria [44]. The results identified higher levels of antibiotic resistance in mesophiles with a greater incidence of multi- and single-drug resistance compared to psychrophiles. Furthermore, the levels of resistance increased with proximity to Palmer Station, which houses a maximum of 46 personnel in the summer (13 in winter) [48]. Two studies specifically screened *Escherichia coli* strains from a range of environmental samples for the presence of AMR at Davis Station (90 people during the summer, 17 in winter) [49, 50]. *E. coli* carrying AMR determinants were isolated from water, sediments and the filter-feeding bivalve, *Laternula elliptica.* Interestingly, no resistance was found in the *E. coli* in the burrowing urchin, *Abatus nimrodi*. This led to the hypothesis that antibiotic-resistant gene spread was being mediated via the water column, which would impact the filter-feeding bivalve more as it actively pumps water across its gills, whereas the urchin feeds on sediment deposits. Clearly, more studies are needed to validate this hypothesis. In a slightly different approach, cultivatable bacteria isolated from three very different areas were screened for antibiotic sensitivity [51]. The first was a remote region of Antarctica, with little recorded human or wildlife activity. The second was an area with known wildlife activity (migratory birds), and the third was an area close to human influence (i.e. research stations). The researchers found some antibiotic resistance in the remote regions and areas associated with wildlife visits, but antibiotic resistance was highest in the human-impacted area. Bacterial diversity varied across the three areas sampled, but the researchers also found an association of resistance to synthetic antibiotics with areas of high anthropogenic activity, leading them to suggest resistance to synthetic antibiotics as a proxy for the human-associated introduction [51]. Similar results associating greater levels of AMR closer to the research stations have also been found in a more recent metagenomics analysis [36]. Thus, in an Antarctic context, AMR in mesophilic bacteria, including *E. coli*, and resistance to synthetic antibiotics have generally been used as proxies for anthropogenic-introduced AMR. This correlative approach is validated by the identification of *E. coli* gene cassettes in Antarctic samples, which are identical to those found in clinical contexts [49].

### Antibiotics and waste management

When research stations were built in Antarctica prior to the 1980s, waste regulation was, at most, minimal. Even the earliest station residents had access to synthetic antibiotics (Additional file 1) with raw sewage introducing antibiotics and human-derived *E. coli* into the ecosystem, albeit at very low levels given the number of stations and occupancy rates in the initial ‘colonisation’ of land south of 60° S. However, regulation of waste disposal and treatment changed dramatically at the end of the twentieth century with the implementation of the Protocol on Environmental Protection to the Antarctic Treaty (also known as the Environmental Protocol or Madrid Protocol). The Protocol was originally signed in 1991 and formally adopted by the signatory Parties in 1998. Annex III to the Environmental Protocol provides strict regulations for minimising and disposing of waste on the continent, including the banning of the introduction of certain products like polychlorinated biphenyls (https://www.ats.aq/e/protocol.html), but its treatment requirements with regard to wastewater disposal are limited to maceration, albeit some stations operate wastewater treatment plants that deliver much higher treatment standards [52, 53]. In 2005, a comprehensive survey of wastewater treatment at Antarctic stations indicated that 37% of the permanent stations and 69% of the summer stations lacked any form of treatment facility [52, 53]. In 2013, the Antarctic Treaty Consultative Meeting adopted the Clean-Up Manual (revised in 2019, available at: https://documents.ats.aq/recatt/Att667_e.pdf) that contained practical guidelines for member states to meet their obligations under Article 1(5) of Annex III to the Protocol.

The introduction of antibiotics and antibiotic-resistant genes into the wider ecosystem is well known via wastewater treatment plants in other global regions [54, 55]. This is also the case in Antarctica, as revealed by five studies, which specifically tested wastewater from research stations [49, 50, 56–58]. These studies characterised the AMR of *E.coli* strains isolated from outfalls of wastewater treatment plants at several sites along the Antarctic Peninsula and South Shetland Islands. They revealed multiple drug resistance to β-lactams, aminoglycosides, tetracycline, and trimethoprim-sulfonamide and included the first identification of a human-associated extended-spectrum β-lactamase (ESBL) in a bacterium isolated from the Antarctic environment [56, 58]. These were followed by a study conducted in 2016–2017, which analysed water samples from the same sites in more detail [57]. In this later study, all samples were analysed for the presence of antibiotics using liquid chromatography coupled to tandem mass spectrometry (LC-MS/MS) and for the presence of antibiotic-resistant *E. coli* [57]. Eight antibiotic compounds were identified in significant quantities using LC-MS/MS, including the quinolones ciprofloxacin and norfloxacin, the macrolides azithromycin, clarithromycin and erythromycin. Whilst antibiotic-resistant *E. coli* were present in the water samples tested for antibiotics, the researchers were unable to directly correlate the observed resistance with the compounds identified in the same samples [57]. Similar evaluations of cultivated antibiotic-resistant *E. coli* collected from various sources around Davis Station also revealed AMR for aminoglycosides and sulphonamides and the presence of mobile genetic element *intl1* amplicons in gene cassettes. These gene cassettes were identical to those found in clinical contexts and thus are almost certainly of human origin [49].

These five isolated studies clearly show incomplete degradation of antibiotics via wastewater treatment plants and their introduction into the Antarctic environment. They also catalogue the persistence of human-derived *E. coli* in very cold waters. These studies highlight the need for more extensive research to determine whether certain types of wastewater treatment plants perform better in such an extreme environment, or if they can be modified to work more efficiently in very cold conditions [53, 57, 59]. Related to this issue of AMR and wastewater treatment is the problem of plastic contamination in Antarctica. In spite of its physical isolation, further enhanced by strong oceanic and atmospheric currents around the continent, various types of plastic waste have been identified in the Southern Ocean, many of which originate from outside of the Antarctic Treaty Area [60]. A recent study investigating the biofilms associated with a piece of macroplastic waste collected from the shores of King George Island identified AMR in associated cultivatable bacteria [61]. This evidence of plastic having the potential to act as a vector of antibiotic-resistant genes represents a novel route for AMR introduction and gives the Antarctic Treaty Consultative Parties a further cause for concern regarding waste management [62].

### AMR in Antarctica: King George Island (a case study)

To date, of all the AMR studies carried out in Antarctica, the majority have been carried out in the South Shetland Islands, particularly King George Island, which is the largest island in the archipelago. The South Shetland Islands are one of the more accessible areas of Antarctica being close to the tip of the Antarctic Peninsula. They also have the greatest density of research stations in Antarctica (20 in total, of which 11 are occupied year-round) (https://www.comnap.aq/). Nine studies concentrated on King George Island and identified both phenotypic and genotypic resistance to a range of natural, semi-synthetic and synthetic antibiotics (Fig. 1, bottom left panel; Additional file 4). Resistance to a range of antibiotic drug classes was found, including β-lactams, macrolides, aminoglycosides, sulphonamides, cephalosporins, polymyxins and fluoroquinolones [30, 39, 47, 51, 56–58, 61, 63]. These studies indicate that resistance to broad-spectrum antibiotics, such as β-lactams and aminoglycosides, is particularly prevalent. This may be due to surveying bias, as nearly all studies investigating phenotypic resistance checked for resistance to β-lactams and aminoglycosides via either antibiotic resistance assays or targeted PCR of antibiotic resistance genes (Fig. 1, bottom right panel; Additional file 5).

Resistance to β-lactams was more common than that to aminoglycosides in most studies carried out in the Antarctic, including King George Island (Additional file 5). Twelve studies found resistance to β-lactams across 29 sites, with phenotypic resistance to ampicillin, penicillin, cefazolin, cefuroxime, methicillin, oxacillin, amoxycillin, penicillin G, carbenicillin, ceftazidime and cefixime identified. Molecular resistance mechanisms to β-lactams, including genes such as bla_CTX-M_, bla_TEM-117_, bla_TEM-157_ and bla_TEM-1_, were also identified [51, 56, 63]. Ten of these studies found resistance to aminoglycosides across 16 sites. Phenotypic resistance was found to aminoglycosides such as streptomycin and kanamycin with the presence of genes such *aadA*, *strA* and *strB* [39, 63]. Additional antibiotic-resistant genes were identified that confer resistance to sulphonamides, e.g. *sul1*, *sul2* and *SulA* [39, 47, 63]; quinolone, e.g. *acra-04*, *oprJ*, *qacedelta* and *qach* [59]; and efflux pumps, e.g. *amrB* and *ceoB* [39]. When the efficacy of some of these genes was assayed, it was interesting to note that the efflux pumps *amrB* and *ceoB* did not increase the tolerance of Antarctic bacteria to levels of clinical concern [39]. Therefore, it was suggested that even if these antibiotic-resistant genes are present, they may require selection pressure to generate significant risk in the future [39].

In terms of how those antibiotic-resistant genes are propagated, the results of the more molecular-centred analyses on King George Island failed to present a clear picture. In general, levels of plasmids and mobile genetic elements were lower in Antarctic bacteria compared with those from other more impacted regions (e.g. samples from active sludge, chicken and swine faeces in Hong Kong and China) [39]. However, one King George Island study identified significant resistance to aminoglycosides, which are often plasmid-encoded [51], whilst another suggested that plasmid-encoded antibiotic-resistant genes were only at low levels, occurring in less than 16% of characterised antibiotic-resistant bacteria [39]. The latter study also suggested that efflux pumps were the major mechanism of AMR in Antarctica, comprising 60% of bacteria showing AMR [39]. This result was supported by shotgun metagenomics data from another region of Antarctica, north of the Mackay Glacier, Victoria Land [32]. These metagenomics data contained many efflux pump sequences indicating the genetic potential for their expression in different Antarctic bacteria but require functional studies for verification of activity. Furthermore, two King George Island studies correlated the presence of specific mobile genetic elements (*Intl1*, *tnp05* and *tnpA2*) with antibiotic-resistant genes. Antibiotic-resistant genes, such as *pikR2*, were associated with the mobile genetic element *Intl1*, whilst *StrB*, *SulA* and *qacedelta1-01* were associated with the transposon *tnpA05* and *SulA*, *qacedelta1-01* and *cmla-01* with transposon *tnpA2* [63]. Interestingly, an earlier study by the same group identified a correlation of the mobile genetic element *Intl1* with the sulphonamide- and quinolone-resistant genes *sul1*, *sul2* and *qnrS*, which was not reproduced in the later study. This may not be surprising as the approaches in the two studies were completely different with PCR of antibiotic-resistant genes directly from soil DNA in one [47] and identified from whole-genome sequencing of cultivated bacteria in the other [63].

Unfortunately, due to the wide variety of methods employed in these studies, it is difficult to develop an overview of AMR incidence on King George Island. For example, five studies surveyed cultivatable bacteria [30, 39, 51, 61, 63] and three specifically screened *E. coli* strains [56–58]. With regard to molecular analysis of antibiotic-resistant genes, targeted PCR was employed in two studies [47, 51], whilst a global metagenomics approach was carried out in a single study [39] (Table 1). Furthermore, the samples ranged from pristine sites to wastewater treatment plants.

**Table 1:** Summary of methods used in King George Island studies

Study	Initial culture media	Antibiotic susceptibility test	Molecular analyses
Hernádez et al. (2012) [56]	ChromoCult® Coliform Agar ES Uniselect4 plates	Disc diffusion: EUCAST	q-RT PCR 3 primer sets Multi-locus sequence typing (MLST)
Tam et al. (2015) [30]	LB, R2A and Marine agar 2216	Disc diffusion using prescience/absence of inhibition	Colony screening by randomly amplified polymorphic DNA (RAPD) analysis and 16S rDNA
Rabbia et al. (2016) [58]	ChromoCult® Coliform Agar ES	Disc diffusion: CLSI	Fingerprint analysis using pulsed field gel electrophoresis (PFGE)
Yuan et al. (2019) [39]	LB media	Disc diffusion: CLSI	Illumina HiSeq 2000 or 2500 100bp PE
Na et al. (2019) [47]	None	None	16S rRNA plus qRT-PCR of 4 ARGs
Hernández et al. (2019) [57]	ChromoCult® Coliform Agar ES	Disc diffusion: CLSI	None
Laganà et al. (2019) [61]	Marine agar 2216	Kirby Bauer test: EUCAST and CLSI	16S rRNA plus restriction fragment length polymorphism (RFLP) analysis
Jara et al. (2020) [51]	R2A agar	Disc diffusion: CLSI	16S rRNA plus PCR of 20 ARGs
Na et al. (2021) [63]	Beef extract peptone agar	Disc diffusion: CLSI	16S rDNA plus qRT-PCR of circa 300 primer sets for ARGs

### Are animal vectors of AMR in Antarctica?

A major gap in our knowledge is the influence of the charismatic megafauna on AMR in the region. Data concerning the role of wildlife as vectors of AMR in Antarctica are very limited, even though it is known that animals and humans may work synergistically to promote the spread of antibiotic-resistant genes [10]. In this context, it is perhaps ironic that there has been considerable interest in the early to mid-twentieth century in characterising the gut flora of polar wildlife, particularly birds [64, 65]. These studies date back to 1899 with the reports of Levin from Spitzbergen and Ekelöf during the Swedish South Polar Expedition in 1901–1903 [64, 66, 67]. The results from these early culture experiments seemed to indicate that the guts of many polar birds were ‘bacteriologically sterile,’ fuelling further research in this area [64]. As culture techniques improved alongside the development of molecular biology, bacteria were readily isolated from bird guts (and occasionally other species, such as fur (*Arctocephalus gazella*) and Weddell seals (*Leptonychotes weddellii*)) [65]. Research then concentrated on the phylogenetic characterisation of serovars via pulsed field gel electrophoresis (PFGE) with a particular interest in identifying *Salmonella* and *Campylobacter* species in Antarctic wildlife, which likely originated from humans or domestic animals (which were allowed on the continent in the early days). These studies provided evidence of reverse zoonosis, with birds such as brown skuas (*Stercorarius antarcticus*) particularly likely to pick up human pathogens as they actively scavenge around areas of human activity [11, 65]. In some of these studies, tests were carried out for phenotypic AMR profiling using standard disc diffusion methods which showed that all *Salmonella* isolates were susceptible [68, 69]. In relatively recent studies of *E. coli* strains found in wastewater outfalls from research stations, low levels of human extra-intestinal *E. coli* strains (ST95 and ST131) were found in seawater, sediments, the clam *Laternula elliptica* and Weddell seals [45, 46]. Furthermore, a study of Weddell seals, fur seals (*Arctocephalus gazella*) and southern elephant seals (*Mirounga leonina*) identified the presence of *E. coli* strains ST73, ST95, ST141 and ST131 in several populations [70]. The transfer of ST95 to endemic wildlife was particularly worrying as this *E. coli* strain is a significant cause of avian disease [49, 50] and also the identification of ST131 in pinnipeds was of concern as ST131 fluoroquinolone-resistant subclones are frequently associated with human urinary tract infections [70]. Thus, for many years since the first colonisations of Antarctica, this limited screening suggested that endemic wildlife was effectively ‘AMR-free’, although there was evidence of increasing levels of reverse zoonosis.

In 2008, the first identification of *tet*(M) resistance was reported in cloacal swabs from Adélie penguins (*Pygoscelis* adeliae) colonising Hukuro Cove, Langhovde, on the coast of eastern Antarctica [71]. The researchers suggested that wildlife may act as reservoirs of antibiotic-resistant genes and spread these through faecal contamination, although the report was very brief and omitted full details of how many penguins were screened. A follow-up study showed that a variety of *tet(M)* genes were present in the same Adélie population [72]. Subsequent studies on animal faeces, representing a total of four emperor (*Aptendytes forsteri*) and almost 450 gentoo penguins (*Pygoscelis papua*), failed to identify antibiotic-resistant bacteria in the faecal samples, with the exception of a single gentoo penguin sample that contained bacterial resistant to chloramphenicol [56, 73, 74]. Such results suggest that antibiotic resistance is rare amongst the bacteria isolated from wild birds in Antarctica. These data are largely supported by other studies. For example, screening of 25 *E. coli* strains collected from cloacal swabs or faecal samples from gentoo penguins, kelp gulls (*Larus dominicanus*) and snowy sheathbills (*Chionis albus*) showed only intermediate level resistance [58]. Three other studies have identified some form of AMR in Antarctic wildlife. A multi-drug resistant strain of *Salmonella enterica* serovar *Enteriditis* was isolated from gentoo penguins close to the Bernardo O’Higgins Research Station on the Antarctic Peninsula in 2014. However, this strain was only found in 2% of penguins sampled and was not found in samples collected in the two subsequent years (2015 and 2016) [75]. The researchers did not determine the source of the drug resistance but hypothesised that the penguins may have carried the resistant pathogen from elsewhere following migration. PCR screening for sulphonamide resistance in faecal samples from single samplings of a penguin, bird and seal in Fildes Peninsula [47] and a single bird dropping from the Fildes Peninsula produced positive results [63]. A further study demonstrated AMR in environmental samples collected from an area designated as having ‘possible animal influence’, but was not correlated with any wildlife sampling [51].

Overall data on AMR in Antarctic wildlife are very limited, with more recent results largely dominated by penguin-derived samples. It is clear that AMR may reside in endemic wildlife, but the extent and transmissibility are virtually unknown. Where instances of AMR in wildlife have been identified, the significance of the findings have been difficult to verify, particularly when they are either not repeatable, or sample numbers are very low. Clearly, more extensive and systematic analyses are required, especially in view of the current COVID-19 pandemic, which has exacerbated concerns about animal-human transmission (and vice versa) of novel viruses and diseases [11, 76].

### Limitations of current data

This review carried out an extensive literature search of articles investigating AMR in Antarctic environments. Analysis in the relevant publications is based on the premise that resistance to synthetic antibiotics is derived from human activity and is largely correlative. There is also the issue of whether culture-based tests for antibiotic activity in the laboratory (often used in medical microbiology) are suitable for AMR detection in this environment, especially as cultivation success levels are low and there may be as yet undefined specific triggers for expression. Overall, the isolated and uncoordinated nature of the studies limits our ability to interpret the results further and estimate the true prevalence of AMR levels on the continent. The results published by many studies describe a few isolates that had been extracted from a limited number of sampling sites, with a variety of different experimental approaches used to describe AMR in terms of antibiotic-resistant bacteria (identified by assaying cultivatable bacteria with antibiotics) or molecular analyses of antibiotic-resistant genes [31, 39, 46, 49]. In some cases, although studies surveyed antibiotic-resistant bacteria in a range of environmental samples (e.g. water, soil and animal faeces), faecal samples where such bacteria were identified were sometimes selected from a single representative animal. Thus, data are too limited to infer the prevalence of AMR in Antarctic animals, especially birds and seals or the role of animals as vectors of AMR in Antarctica [47, 63, 77]. In addition, there are few current data, with many studies published years after the sample collection dates. In this synthesis, the most recent sampling date was 2019, with most studies falling before 2015 [28, 30, 33, 58, 73]. Furthermore, many studies did not report the exact sampling locations in a systematic manner. In some instances, both the named area and coordinates were reported, whilst others merely named an area or provided maps indicating sampling points [32, 41, 46, 47, 63, 73]. Reporting precise coordinates of sampling sites in conjunction with maps or site names should be standard, as it provides uniformity that can support comparative analyses.

Culture-based approaches for investigating antibiotic resistance were employed by most studies. Whilst these are useful for associating resistance with a particular bacterium, they do not provide data on the molecular resistance mechanisms unless whole-genome sequencing is employed. Very few antibiotic-producing bacteria are currently cultivatable in the laboratory, which restricts the capacity to fully capture antibiotic-resistant gene diversity or abundance, unless deep sequencing metagenomics approaches are employed [32, 39]. In addition, some studies screened the isolated bacteria for phenotypic antibiotic resistance, but a uniform definition of phenotypic resistance was absent. Most studies quoted the use of the Clinical and Laboratory Standards Institute (CSLI) guidelines [35, 39, 51, 58, 74]. However, one study determined phenotypic resistance using antibiotic discs and determined the absence of bacterial growth around the disc as susceptibility [30]. Similarly, another study mentioned using the Kirby-Bauer test, but not the use of CSLI guidelines [61]. A further study cultivated bacteria on Muller Hinton agar with concentrations of antibiotic according to WHO [78, 79] and German (DIN Norm 58930) standards [42].

### Future prospects

It is clear from the research carried out to date that more extensive and detailed research is needed to obtain even a reasonable understanding of the extent of AMR in Antarctica and the influence of humans in this regard. Given the chronic and generally accumulative nature of the AMR problem, any short-term evaluations need to be conducted alongside long-term monitoring of AMR in designated regions, specifically at/near research stations. More extensive surveys are also required of the AMR status of endemic wildlife to determine how they may act as either reservoirs of AMR and/or vectors of AMR (of either natural or anthropogenic origin) and the occurrence of reverse zoonosis [11]. In parallel, a greater standardisation of approach is needed to enable direct comparisons between different studies [80].

Greater emphasis needs to be placed on reporting metadata, with minimal reporting standards introduced, including logging the exact sampling date and GPS co-ordinates for each sampling event. Studies which combine both culture-based and metagenomics and genome sequencing approaches are to be encouraged, as only then can real links be made between AMR, the underlying mechanisms and how antibiotic resistance genes are propagated in this relatively pristine environment (i.e. the prevalence and role of plasmids and mobile genetic elements). Similarly, sequencing approaches should enable better differentiation between natural/intrinsic and anthropogenically introduced/acquired AMR and also shed light on the role of animals in AMR interactions. With regard to culture-based methodologies, there is a requirement for an equivalent to EUCAST/CLSI breakpoints for environmental bacteria. Currently, there are no standardised breakpoints to validate disc diffusion assays with non-clinical bacterial isolates. In this regard, minimal inhibitory concentration (MIC) can be used to study phenotypic resistance. However, with many samples and multiple antimicrobials to test, this is a costly and laborious task. These recommendations should form the basis for discussion and the development of an AMR protocol and checklist by Antarctic Treaty Consultative Parties, as discussed further below.

Due to the extreme environmental conditions, many Antarctic terrestrial environments are dominated by microbial species, and these comparatively simple communities provide excellent opportunities for research that would be more difficult elsewhere. For some time, researchers have recognised the importance of reducing the release of human-associated microorganisms into marine and terrestrial environments to prevent ‘genetic pollution’ of the continent [12, 49, 81, 82]. The Antarctic Treaty Consultative Meeting has taken steps to reduce microbial contamination of a small number of areas through the designation of ‘restricted’ zones within some Antarctic Specially Protected Areas (ASPAs) (e.g. ASPA 126 Byers Peninsula, Livingstone Island) [81]. Furthermore, the Scientific Committee on Antarctic Research (SCAR) has developed a series of field codes of conduct (https://www.scar.org/policy/scar-codes-of-conduct/), which are endorsed by the ATCM, that include recommendations to reduce contamination of terrestrial and geothermal environments by microorganisms originating from human sources and other environments. However, much still needs to be done with the issue of AMR having been little discussed within the Antarctic Treaty System [83]. With maceration, the only treatment mandated by the Environmental Protocol, and many stations (particularly the small stations or those occupied only during the summer months) providing little or no sewage treatment, there is scope for substantial improvement in standards, which may reduce the release of antibiotics, human-derived *E. coli* and antibiotic-resistant bacteria into the marine and/or ice environment [82]. The unique governance structure of the Antarctic Treaty region provides an opportunity to enact continent-wide regulations and standards on AMR monitoring, which would not only encourage long-term monitoring, but also provide input into waste management strategies and insight into AMR levels (endemic and human-introduced) in this remote region.

### Summary

Although traditionally viewed as a relatively pristine environment, antibiotic-resistant genes and antibiotic-resistant determinants were found in studies all across the continent. Initial evaluations suggest that AMR in most of Antarctica may closely represent a ‘pre-antibiotic state’ [39] and can therefore be used as a proxy to model AMR interactions and propagation in more industrialised regions. However, many questions remain, especially with regard to the levels of naturally occurring AMR (as opposed to anthropogenically introduced AMR) due to the isolated, ad hoc nature of the studies reported and the wide varieties of methodologies employed. Certainly, more functional studies are required to verify the activities and interactions of antibiotic resistance genes identified in sequencing studies, along with whole-genome sequencing of cultivated Antarctic antibiotic-resistant bacteria. The current data clearly indicate that there is much potential for the future in research evaluating AMR in Antarctica, which may not be as pristine with regard to AMR as previously assumed, particularly around research stations. In addition, there are clear policy outcomes from such studies with regard to wastewater management in the region. Finally, whilst this review describes the current state of knowledge concerning AMR in Antarctica, this extreme region is also viewed as a potential source of novel compounds, including antimicrobial agents [84–86], which may well be uncovered in future AMR evaluations, particularly those focussed on sequencing approaches.

## Supplementary Information

**Additional file 1.** Extract from MV John Biscoe medical stores list written by Dr William Sladen in 1949, when he was the doctor on call between postings at Hope Bay (Antarctic Peninsula) and Signy Island (South Orkney Islands), showing tablets and bottles of sulphathiazole.

**Additional file 2.** Initial screening criteria for literature review. Parameters for choice of studies investigating AMR on the Antarctic continent.

**Additional file 3.** Referenced GPS locations of studies of AMR in Antarctica.

**Additional file 4.** Summarised data of studies on King George Island.

**Additional file 5.** Locations of studies that found resistance markers for β-lactams and aminoglycosides in Antarctica.

**Additional file 6.** Natural, synthetic and semi-synthetic antibiotics and their drug classes. Examples of antibiotics, the drug classes they belong to, their targets and nature (natural, semi-synthetic or synthetic). The list is not exhaustive of all antibiotics or drug classes.

## Data Availability

Not applicable

## Notes

Declarations

## Notes

Ethics approval and consent to participate

## Notes

Consent for publication
