# Supplementary material for: Antimicrobial resistance in Antarctica: is it still a pristine environment?
Source: Microbiome. 2022 May 6;10:71. doi: 10.1186/s40168-022-01250-x (PMC9072757; doi:10.1186/s40168-022-01250-x)

**Additional File 1:** Extract from MV John Biscoe medical stores list written by Dr William Sladen in 1949, when he was the doctor on call between postings at Hope Bay (Antarctic Peninsula) and Signy Island (South Orkney Islands), showing tablets and bottles of sulphathiazole.


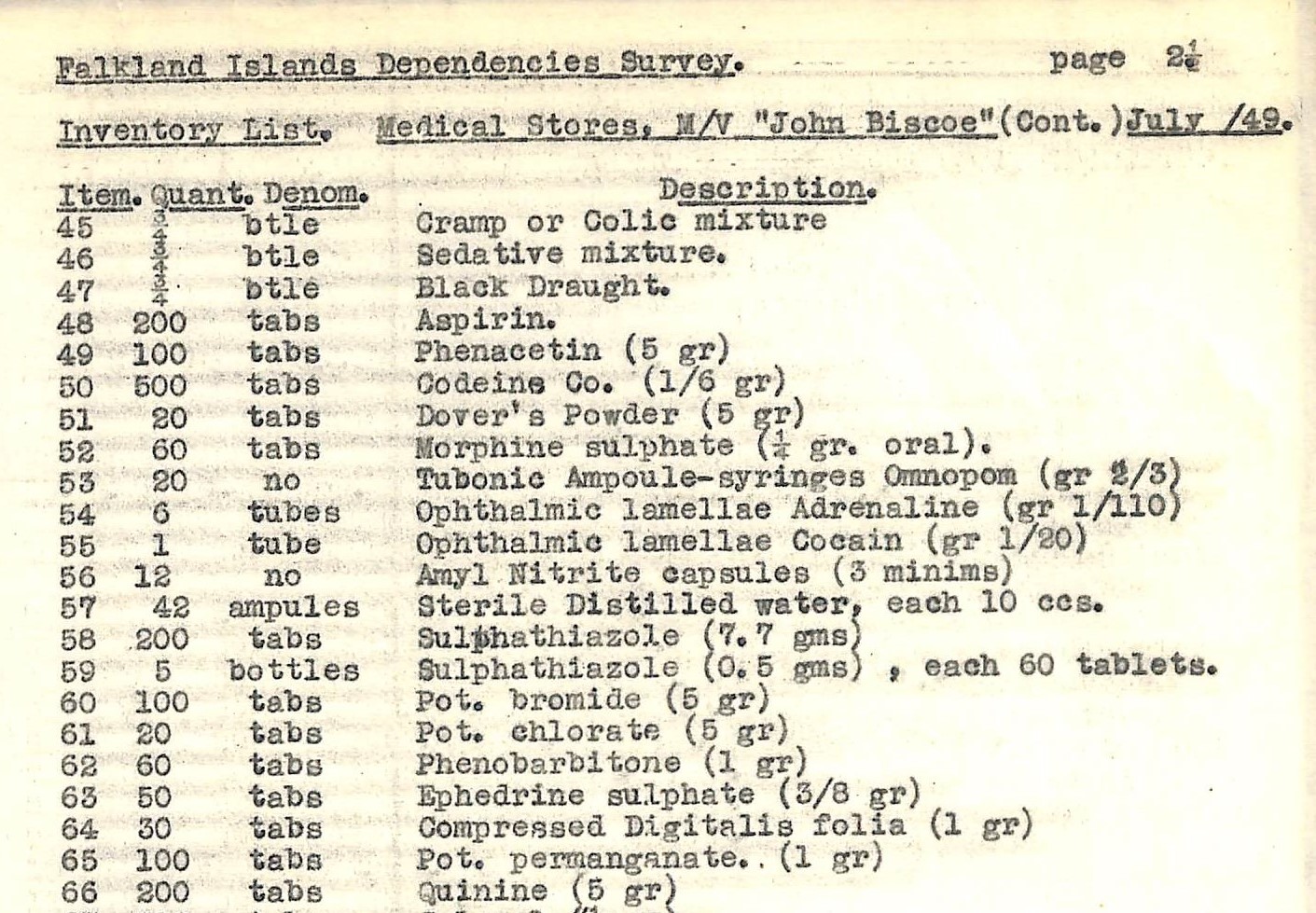

Supplement: Supplementary file 2 — Additional file 1. Extract from MV John Biscoe medical stores list written by Dr William Sladen in 1949, when he was the doctor on call between postings at Hope Bay (Antarctic Peninsula) and Signy Island (South Orkney Islands), showing tablets and bottles of sulphathiazole. [file 40168_2022_1250_MOESM1_ESM.docx]
