# Supplementary material for: Antimicrobial resistance in Antarctica: is it still a pristine environment?
Source: Microbiome. 2022 May 6;10:71. doi: 10.1186/s40168-022-01250-x (PMC9072757; doi:10.1186/s40168-022-01250-x)
Supplement: Supplementary file 3 — Additional file 2. Initial screening criteria for literature review. Parameters for choice of studies investigating AMR on the Antarctic continent. [file 40168_2022_1250_MOESM2_ESM.docx]

**Additional File 2: Initial screening criteria for literature review:**

The terms ‘antimicrobial resistance’ or ‘antibiotic resistance’ and ‘Antarctica’ were queried into PubMed (https://pubmed.ncbi.nlm.nih.gov/) with a cut-off date of October 2021, with a further revision in Jan 2022. Antarctica was defined according to the Antarctic Treaty System as the area south of a latitude of 60˚. All papers that screened for ARGs, ARBs, or resistance-associated plasmids on the Antarctic continent were included in this study, although studies screening Antarctic bacteria for novel antimicrobial peptides and metabolites for potential biotechnology exploitation were specifically excluded. The Table below details the inclusion and exclusion criteria for each study. The results of the initial screen often led to additional literature of associated studies, which did not necessarily include the relevant keywords.

**Table: Parameters for choice of studies investigating AMR on the Antarctic continent**

| **Parameter** | **Inclusion Criteria** | **Exclusion Criteria** |
| --- | --- | --- |
| Location | Latitude south of 60˚ | Latitude north of 60˚ |
| Type of data | Antibiotic resistance genes, antibiotic resistance bacteria, resistance-associated plasmids | Metal resistance  Microbial studies of staff working on station  Bioprospecting  Biomining  Novel antimicrobial peptides/metabolites |
| Type of sample | Soil, water, glacier, animal faeces, snow, non-living material isolated from the environment, Antarctic organisms | Not specified |
