# Supplementary material for: Antimicrobial resistance in Antarctica: is it still a pristine environment?
Source: Microbiome. 2022 May 6;10:71. doi: 10.1186/s40168-022-01250-x (PMC9072757; doi:10.1186/s40168-022-01250-x)
Supplement: Supplementary file 4 — Additional file 3. Referenced GPS locations of studies of AMR in Antarctica. [file 40168_2022_1250_MOESM3_ESM.docx]

**Additional File 3: Referenced GPS locations of studies of AMR in Antarctica**. The GPS coordinates differ in format between studies due to differences in reporting. The locations of the studies highlighted in grey are where the authors did not provide an exact sample site, but rather plotted their location onto a figure. The GPS coordinates are therefore those close to the identified sampling area or the track of the cruise. Where there are two latitudes and longitudes against a study, this is where numerous samples were collected across a range and the limits of the study are defined.

| **Author** | **Latitude** | **Longitude** | **Author** | **Latitude** | **Longitude** |
| --- | --- | --- | --- | --- | --- |
| **Kobori, Sullivan and Shizuya, 1984** | 77.8590°S | 166.6897°E | **Mora et al, 2018** | 62°15’S | 58°37’W |
| **Kobori, Sullivan and Shizuya, 1984** | 77.6000°S | 163.8500°E |  | 67°46’S | 68°43W |
| **De Souza et al, 2007** | 70°45′30″S | 11°38′40″E | **Pantůček et al, 2018** | 63°48'51"S | 57°50'45"W |
| **Lo Giudice et al, 2007** | 74°41’33’’S | 164°07’15’’E | **Cerdà-Cuéllar et al, 2019** | 62° 36′ 0″ S | 60° 30′ 0″ W |
| **Lo Giudice et al, 2007** | 74°43’S | 164°16’E | **Yang et al, 2019** | 64°46'12.0"S | 64°03'00.0"W |
| **Bonnedahl et al, 2008** | 64°50′S | 062°33′W |  | 75.7559°S | 168.2359°E |
| **Rahman et al, 2008** | 69°13′S | 039°39′ E | **Ghaly et al, 2019** | 66°24’36.76”S | 110°39’17.40”E |
| **Rahman et al, 2015** | 69°13′S | 039°39′ E | **Yuan et al , 2019** | 62°15.910S | 58°52.738W |
| **Miller, Gammon and Day, 2009** | 64°10′S | 61°50′W |  | 62°13.200S | 58°57.85W |
| **Ushida et al, 2010** | 63° 55′ S | 58° 10′ W | **Hernández et al, 2019** | 62º 12’1.65”S | 58º 57’36.96”W |
| **Timmery, Hu and Mahillon, 2011** | 75°05′59″S | 123°19′56″E | **Hernández et al, 2019** | 62° 12’5.07”S | 58° 57’39.58”W |
| **Vigo et al, 2011** | 62° 15′ 0″S | 58° 40′ 0″ W | **Hernández et al, 2019** | 62° 11’59.37”S | 58 °57’31.16”W |
| **Vigo et al, 2011** | 63° 23′ 0″S | 56° 59′ 0″ W | **Okubo et al, 2019** | 77°19′S | 39°42′E |
| **Hernández et al, 2012** | 63°19’15’’S | 57°53’55’’W | **Laganà et al, 2019** | 62° 11′ 53.5” S | 058° 56′ 29.6″ W |
| **Hernández et al, 2012** | 62°28’44’’S | 59°39’52’’W | **Antelo et al, 2021** | 62°0903”’S | 58°56’27”W |
| **Lo Giudice et al, 2013** | 74°41.698′S | 164°04′214″E |  | 78°01’23”S | 164°06’02”W |
| **Lo Giudice et al, 2013** | 74°41′80.3″S | 164°07′80.3″E | **Hernández et al, 2019** | 62º 12’1.65”S | 58º 57’36.96”W |
| **Segawa et al, 2012** | 69°03’S | 40°41’E | **Hernández et al, 2019** | 62° 12’5.07”S | 58° 57’39.58”W |
|  | 80°58’S | 82°45’W | **Hernández et al, 2019** | 62° 11’59.37”S | 58 °57’31.16”W |
| **Segawa et al, 2013** | 69°03′S | 40°41′E | **Okubo et al, 2019** | 77°19′S | 39°42′E |
|  | 80°58′S | 82°45′W | **Laganà et al, 2019** | 62° 11′ 53.5” S | 058° 56′ 29.6″ W |
| **Gunnigle et al, 2015** | 78°60′S | 164°0′E | **Miwa et al, 1976** | 69°00′16″S | 39°34′54″E |
| **Tam et al, 2015** | 62°12′14.9″S | 58°57′47.5''W | **Wynn-Williams, 1983** | 54°14’ - 67°34’ | Not stated |
| **Tam et al, 2015** | 62°58′56.3″S | 60°39′51.1″W | **Olsen et al, 1996** | 54°0'0"S | 38°2'59"W |
| **Power et al, 2016** | 68°.35′S | 77°58′ E | **Palmgren et al, 2000** | 54°0’0”S | 38°2’59”W |
| **Stark et al, 2016** | 68.5764°S | 77.9689° E | **De Souza et al, 2006** | 50°S - 65°S | 18°E - 30°E |
| **Wang et al, 2016** | 74°37’26”S | 164°13’44”E | **Skurnik et al, 2006** | Not stated | Not stated |
| **Rabbia et al, 2016** | 62°12′1.65″S | 58°57′36.96″W | **Van Goethem et al, 2018** | 76°58’S | 162°0’E |
|  | 64°49′26.03″S | 62°51′26.10″W | **Na et al, 2019** | 62°12’59.70”S | 58°57’51.90”W |
| **Retamal et al, 2017** | 62°13′S | 58°56′W | **Blanco-Picazo et al, 2020** | 62°36’S | 60°30’W |
|  | 64°49′26″S | 62°51′25″W | **Jara et al, 2020** | 62°12’59.70”S | 58°57’51.90”W |
|  | | | **Na et al, 2021** | 62°12’59.70”S | 58°57’51.90”W |
