# Supplementary material for: Antimicrobial resistance in Antarctica: is it still a pristine environment?
Source: Microbiome. 2022 May 6;10:71. doi: 10.1186/s40168-022-01250-x (PMC9072757; doi:10.1186/s40168-022-01250-x)
Supplement: Supplementary file 5 — Additional file 4. Summarised data of studies on King George Island. [file 40168_2022_1250_MOESM4_ESM.docx]

**Additional File 4: Summarised data of studies on King George Island.** The table shows the locations of AMR studies on King George Island, including details of the class of antibiotics resistance was found to, the antibiotic names and their nature, i.e., whether they are natural, semi-synthetic or synthetic antibiotics. The GPS coordinates differ in format between studies due to differences in reporting. Code to colours: red: synthetic resistance; yellow: semi-synthetic; red: synthetic; green: natural and semi-synthetic. No locations with (i) natural resistance, (ii) natural and synthetic or (iii) synthetic and semi-synthetic combinations were found.

| **Author** | **Latitude** | **Longitude** | **Antibiotic Class** | **Antibiotic** | **Antibiotic Nature** |
| --- | --- | --- | --- | --- | --- |
| **Tam et al, 2015** | 62°12′14.9″S | 58°57′47.5''W | β-lactam, aminoglycoside, cephalosporin, fluoroquinolone, lincosamide, macrolide, aminocoumarin, sulfonamide, glycopeptide antibiotic, quinolone, polymyxin and broad-spectrum antibiotics | Carbenicillin, ceftazidime, cefixime, cefpirome, cephalothin, ciprofloxacin, clarithromycin, clindamycin, erythromycin, fusidic acid, gentamicin, latamoxef, lincomycin, metronidazole, mupirocin, nitrofurantoin, novobiocin, rifampicin, spectinomycin, sulfonamides compound, trimethoprim, vancomycin, ampicillin, nalidixic acid, chloramphenicol, kanamycin sulfate, tetracycline hydrochloride, and polymyxin B sulfate | Natural,  Semi-synthetic  and Synthetic |
| **Yuan et al, 2019** | 62°15.910′S | 58°52.738′W | Possible ARG-carrying plasmids for multidrug resistance and chloramphenicol | Possible ARG-carrying plasmids for multidrug resistance and chloramphenicol | Semi-synthetic |
| **Yuan et al, 2019** | 62°13.021S | 58°57.741W | Possible ARG-carrying plasmids for multidrug resistance and chloramphenicol | Possible ARG-carrying plasmids for multidrug resistance and chloramphenicol | Semi-synthetic |
| **Yuan et al, 2019** | 62°12.666S | 58°55.487W | Possible ARG-carrying plasmids for multidrug resistance and chloramphenicol | Possible ARG-carrying plasmids for multidrug resistance and chloramphenicol | Semi-synthetic |
| **Yuan et al, 2019** | 62°13.710S | 58°55.487W | Possible ARG-carrying plasmids for multidrug resistance and chloramphenicol | Possible ARG-carrying plasmids for multidrug resistance and chloramphenicol | Semi-synthetic |
| **Yuan et al, 2019** | 62°12.667S | 58°55.65W | Macrolide, rifampin, oxazolidinone and β-lactam | Erythromycin, rifampin, ampicillin, penicillin and cefazolin | Natural and Semi-synthetic |
| **Yuan et al, 2019** | 62°12.867S | 58°55.867W | Macrolide, rifampin, oxazolidinone and β-lactam | Erythromycin, rifampin, ampicillin, penicillin and cefazolin | Natural and Semi-synthetic |
| **Yuan et al, 2019** | 62°12.200S | 58°59.75W | Macrolide, rifampin, oxazolidinone and β-lactam | Erythromycin, rifampin, ampicillin, penicillin and cefazolin | Natural and Semi-synthetic |
| **Yuan et al, 2019** | 62°13.200S | 58°57.85W | Macrolide, rifampin, oxazolidinone and β-lactam | Erythromycin, rifampin, ampicillin, penicillin and cefazolin | Natural and Semi-synthetic |
| **Hernández et al, 2012** | 63°19’15’’S | 57°53’55’’W | β-lactam and broad-spectrum antibiotics | tetracycline, ampicillin, streptomycin, chloramphenicol, nalidixic acid, cefadroxil, fosfomycin, tigecycline, trimethoprim-sulfamethoxazole, nitrofurantoin, amdinocillin | Natural,  Semi-synthetic and  Synthetic |
| **Hernández et al, 2019** | 62º 12’1.65”S | 58º 57’36.96” W | β-lactam, aminoglycoside, tetracycline, trimethroprim, sulfonamide and quinolone | Ampicillin, tetracycline, trimethoprim, sulfonamide, cefoxitin, streptomycin, nalixidic acid and cefotaxime | Natural,  Semi-synthetic and  Synthetic |
| **Hernández et al, 2019** | 62º 12’5.07”S | 58º 57’39.58” W | β-lactam, aminoglycoside, tetracycline, trimethroprim, sulfonamide and quinolone | Ampicillin, tetracycline, trimethoprim, sulfonamide, cefoxitin, streptomycin, nalixidic acid and cefotaxime | Natural,  Semi-synthetic and  Synthetic |
| **Hernández et al, 2019** | 62º 11’59.37”S | 58 º57’31.16” W | β-lactam, aminoglycoside, tetracycline, trimethroprim, sulfonamide and quinolone | Ampicillin, tetracycline, trimethoprim, sulfonamide, cefoxitin, streptomycin, nalixidic acid and cefotaxime | Natural,  Semi-synthetic and  Synthetic |
| **Laganà et al, 2019** | 62° 11′ 53.5”S | 058° 56′ 29.6″ W | β-lactam, cephalosporin, macrolide, fosfomycin, oxazolidinone, glycopeptide inhibitor, quinolone and phenicol derivative | Cefuroxime, erythromycin, fosfomycin, lincomycin, linezolid, methicillin, oxacillin, penicillin, teicoplanin, vancomycin, amoxycillin, azithromycin, carbenicillin, cefazolin, cinoxacin, chloramphen and mezlocillin, | Natural and Semi-synthetic |
| **Rabbia et al, 2016** | 62°12′1.65″S | 58°57′36.96″W | β-lactam, cephalosporin, aminoglycoside, quinolone, broad-spectrum antibiotics, sulofnamide and trimethroprim | Ampicillin, cephalothin, cefoxitin, cefotaxime, ceftazidime, cefepime, streptomycin, gentamicin, nalidixic acid, ciprofloxacin, tetracycline, sulfonamide and trimethroprim | Natural,  Semi-synthetic and  Synthetic |
| **Rabbia et al, 2016** | 62°12′5.07″S | 58°57′39.58″W | β-lactam, cephalosporin, aminoglycoside, quinolone, broad-spectrum antibiotics, sulofnamide and trimethroprim | Ampicillin, cephalothin, cefoxitin, cefotaxime, ceftazidime, cefepime, streptomycin, gentamicin, nalidixic acid, ciprofloxacin, tetracycline, sulfonamide and trimethroprim | Natural,  Semi-synthetic and  Synthetic |
| **Rabbia et al, 2016** | 62°11′59.37″S | 58°57′31.16″W | β-lactam, cephalosporin, aminoglycoside, quinolone, broad-spectrum antibiotics, sulofnamide and trimethroprim | Ampicillin, cephalothin, cefoxitin, cefotaxime, ceftazidime, cefepime, streptomycin, gentamicin, nalidixic acid, ciprofloxacin, tetracycline, sulfonamide and trimethroprim | Natural,  Semi-synthetic and  Synthetic |
| **Rabbia et al, 2016** | 62°12′58.65″S | 58°57′35.21″W | β-lactam, cephalosporin, aminoglycoside, quinolone, broad-spectrum antibiotics, sulofnamide and trimethroprim | Ampicillin, cephalothin, cefoxitin, cefotaxime, ceftazidime, cefepime, streptomycin, gentamicin, nalidixic acid, ciprofloxacin, tetracycline, sulfonamide and trimethroprim | Natural,  Semi-synthetic and  Synthetic |
| **Jara et al, 2020** | 62°12’59.70”S | 58°57’51.90”W | β-lactam, aminoglycoside, quinolone, and cephalosporin | Ampicillin, cefalotin, cefuroxime, cefotaxime, ceftazidime, cefepime, streptomycin, kanamycin, amikacin, gentamicin, nalidixic acid, ciprofloxacin, tetracycline, chloramphenicol | Natural,  Semi-synthetic and  Synthetic |
| **Na et al, 2019** | 62°12’59.70”S | 58°57’51.90”W | Sulphonamide and fluoroquinolone | Detected sul1, sul2 and qnrs in some samples | Synthetic |
| **Na et al, 2021** | 62°12’59.70”S | 58°57’51.90”W | Sulphonamide and fluoroquinolone | - | Synthetic |
