# Supplementary material for: Antimicrobial resistance in Antarctica: is it still a pristine environment?
Source: Microbiome. 2022 May 6;10:71. doi: 10.1186/s40168-022-01250-x (PMC9072757; doi:10.1186/s40168-022-01250-x)
Supplement: Supplementary file 6 — Additional file 5. Locations of studies that found resistance markers for β-lactams and aminoglycosides in Antarctica. [file 40168_2022_1250_MOESM5_ESM.docx]

**Additional File 5: Locations of studies that found resistance markers for β-lactams and aminoglycosides in Antarctica.** The GPS coordinates differ in format between studies due to differences in reporting. Colour code: orange: studies which detected resistance to β-lactams only; blue: resistance to aminoglycosides only and green: resistance to both aminoglycosides and β-lactams.

| **Author** | **Latitude** | **Longitude** |
| --- | --- | --- |
| **Kobori, Sullivan and Shizuya, 1984** | 77.8590°S | 166.6897°E |
| **Kobori, Sullivan and Shizuya, 1984** | 77.6000°S | 163.8500°E |
| **Hernández et al, 2012** | 63°19’15’’S | 57°53’55’’W |
| **Hernández et al, 2012** | 62°28’44’’S | 59°39’52’’W |
| **Pantůček et al, 2018** | 63°48'51"S | 57°50'45"W |
| **Yuan et al, 2019** | 62°12.667S | 58°55.65W |
| **Yuan et al, 2019** | 62°12.867S | 58°55.867W |
| **Yuan et al, 2019** | 62°12.200S | 58°59.75W |
| **Yuan et al, 2019** | 62°13.200S | 58°57.85W |
| **Yang et al, 2019** | 64°46'12.0"S | 64°03'00.0"W |
| **Yang et al, 2019** | 64°46'12.0"S | 64°03'00.0"W |
| **Yang et al, 2019** | 64°46'12.0"S | 64°03'00.0"W |
| **Yang et al, 2019** | 75.7529°S | 168.7595°E |
| **Yang et al, 2019** | 75.4580°S | 168.997°E |
| **Yang et al, 2019** | 75.7559°S | 168.2359°E |
| **Yang et al, 2019** | 74.8002°S | 169.3836°E |
| **Laganà et al, 2019** | 62° 11′ 53.5”S | 058° 56′ 29.6″W |
| **Blanco-Picazo et al, 2020** | See Fig. S4 | See Fig. S4 |
| **Power et al, 2016** | 68°.35′ S | 77°58′ E |
| **Stark et al, 2016** | 68.5764° S | 77.9689° E |
| **Okubo et al, 2019** | 77°19′S | 39°42′E |
| **De Souza et al, 2006** | 50°S-65°S | 18°E-30°E |
| **De Souza et al, 2007** | 70°45′30″S | 11°38′40″E |
| **Lo Giudice et al, 2013** | 74°41.698′S | 164°04′214″E |
| **Lo Giudice et al, 2013** | 74°41′80.3″S | 164°07′80.3″E |
| **Tam et al, 2015** | 62°12′14.9″S | 58°57′47.5''W |
| **Tam et al, 2015** | 62°58′56.3″S | 60°39′51.1″W |
| **Rabbia et al, 2016** | 62°12′5.07″S | 58°57′39.58″W |
| **Rabbia et al, 2016** | 62°11′59.37″S | 58°57′31.16″W |
| **Rabbia et al, 2016** | 62°12′58.65″S | 58°57′35.21″W |
| **Retamal et al, 2017** | 63°19′15″S | 57°53′59″W |
| **Hernández et al, 2019** | 62º 12’1.65”S | 58º 57’36.96”W |
| **Hernández et al, 2019** | 62º 12’5.07”S | 58º 57’39.58”W |
| **Hernández et al, 2019** | 62º 11’59.37”S | 58 º57’31.16”W |
