# Supplementary material for: Antimicrobial resistance in Antarctica: is it still a pristine environment?
Source: Microbiome. 2022 May 6;10:71. doi: 10.1186/s40168-022-01250-x (PMC9072757; doi:10.1186/s40168-022-01250-x)
Supplement: Supplementary file 7 — Additional file 6. Natural, synthetic and semi-synthetic antibiotics and their drug classes. Examples of antibiotics, the drug classes they belong to, their targets and nature (natural, semi-synthetic or synthetic). The list is not exhaustive of all antibiotics or drug classes. [file 40168_2022_1250_MOESM6_ESM.docx]

**Additional File 6: Natural, synthetic and semi-synthetic antibiotics and their drug classes**. Examples of antibiotics, the drug classes they belong to, their targets and nature (natural, semi-synthetic or synthetic). The list is not exhaustive of all antibiotics or drug classes.

| **Antibiotic** | **Drug Class** | **Nature** | **Target** |
| --- | --- | --- | --- |
| **Ampicillin** | β-lactam | Semi-synthetic | Cell wall |
| **Azithromycin** | Macrolide | Semi-synthetic | 50S ribosome |
| **Chloramphenicol** | Amphenicol | Semi-synthetic | 50S ribosome |
| **Ciprofloxacin** | Quinolone | Synthetic | DNA gyrase |
| **Clindamycin** | Lincosamide | Semi-synthetic | 50S ribosome |
| **Doxycline** | Tetracycline | Semi-synthetic | 30S ribosome |
| **Erythromycin** | Macrolide | Natural | 50S ribosome |
| **Fosfomycin** | Fosfomycin | Natural | Cell wall |
| **Fusidic acid** | Fusidanes | Natural | 50S ribosome |
| **Gentamycin** | Aminoglycosides | Natural | 30S ribosome |
| **Kanamycin** | Aminoglycosides | Natural | 30S ribosome |
| **Lincomycin** | Lincosamide | Natural | 50S ribosome |
| **Linezolid** | Oxazolidinone | Synthetic | 50S ribosome |
| **Mupirocin** | Mupirocin | Natural | RNA synthetase |
| **Penicillin** | β-lactam | Natural | Cell wall |
| **Polymyxin** | Colistin | Natural | Cell membrane |
| **Rifampicin** | Rifamycin | Semi-synthetic | mRNA transcription |
| **Streptomycin** | Aminoglycosides | Natural | 30S ribosome |
| **Sulfamethoxazole** | Sulfonamide | Synthetic | Nucleic acid synthesis |
| **Tetracycline** | Tetracycline | Natural | 30S ribosome |
| **Vancomycin** | Glycopeptide | Natural | Cell wall |
